# Microscopic Imaging Technology Assisted Dynamic Monitoring and Restoration of Micron-Level Cracks in the Painted Layer of Terracotta Warriors and Horses of the Western Han Dynasty

**DOI:** 10.3390/polym14040760

**Published:** 2022-02-15

**Authors:** Juanli Wang, Jiaxin Li, Xiaolian Chao, Youlu Chen, Yongsheng Huang, Bingjie Mai, Yuhu Li, Jing Cao

**Affiliations:** 1 Engineering Research Center of Historical and Cultural Heritage Protection, Ministry of Education, School of Materials Science and Engineering, Shaanxi Normal University, Xi’an 710119, China; wangjuanli@snnu.edu.cn (J.W.); tuoyuanxinggungun@snnu.edu.cn (J.L.); chaoxl@snnu.edu.cn (X.C.); 2 Cultural Relics Restoration Department, Xianyang Museum, Xianyang 712000, China; skycyl666@163.com (Y.C.); huangyongsheng69@163.com (Y.H.)

**Keywords:** terracotta warriors and horses, micron cracks of the painted layer, microscopic imaging technology, dynamic monitoring, restoration

## Abstract

Cracks are one of the most common issues affecting colored pottery relics; these can be divided into macroscopic cracks, recognizable by the human eye, and micron cracks, which cannot be observed by the naked eye. The gradual development of micron cracks eventually leads to large-scale cracks and the shedding of the coating layer. The repair of such micron cracks poses a key technical difficulty in restoring painted pottery remnants from the Western Han Dynasty. We attempt to solve this problem by reporting on a method that entails the use of a water-borne fluoropolymer material as the adhesive agent, as well as ultra-depth-of-field, digital microscopic imaging technology to build an operating platform for an optical imaging monitoring system. By making simulated ceramic samples, we systematically investigated the influences of water-borne fluoropolymer on chromaticity, adhesion, contact angle, surface morphology, and thermal stability of the paint layer. The results indicate that the color of the painted layer, when treated with the water-borne fluoropolymer, did not change, and the adhesion and contact angle of the painted layer were improved. Additionally, the outcomes of the SEM analysis show that the adhesion and hydrophobicity of the painted layer were improved because the water-borne fluoropolymer filled up the porous structure of the painted layer and covered the pigment particles. These findings demonstrate that aqueous, water-borne fluoropolymer can be used as an adhesive agent for micron cracks. Meanwhile, via the operating platform of the optical imaging monitoring system, the micron cracks of the painted terracotta warriors and horses from the Western Han Dynasty were successfully repaired using the water-borne fluoropolymer. The results imply that the microstructure, size, and geometric spaces of the cracks can be obtained directly utilizing microscopic imaging technology. The dynamic monitoring and imaging system described above can be employed to assist prosthetists in visualizing micro-repair operations in real time, assist with fine visual operations during the repair process, and realize dynamic video recording of the entire repair process. Our work provides a simple visualization method to repair micron-scale cracks in painted pottery relics by applying modern fluoropolymer and ultra-depth-of-field digital microscopic imaging technology.

## 1. Introduction

**Figure 1:**
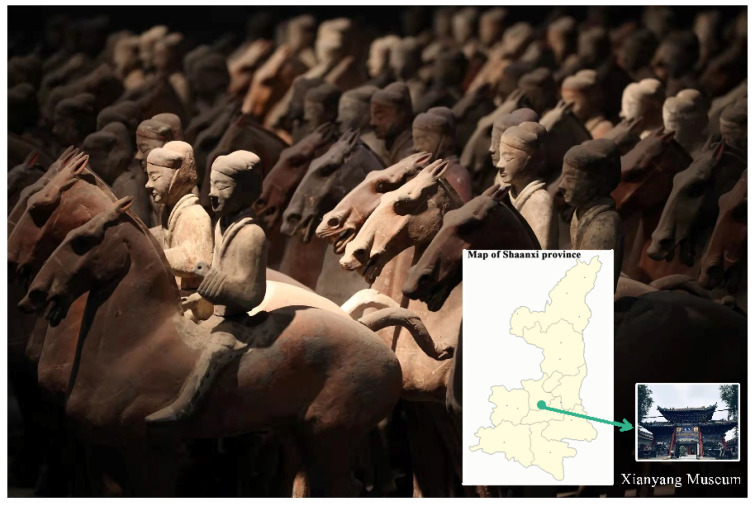
Location of the painted terracotta warriors and horses of the Western Han Dynasty in Yangjiawan, Shaanxi.

The painted terracotta warriors unearthed from the Western Han Dynasty are mainly concentrated in three places: Yangjiawan [1,2], the Hanyang Mausoleum [3,4] in Shaanxi, and at the western foot of Lion Mountain in Yunlong District in the city of Xuzhou [5]. They provide important information for research on the society, culture, art, and customs of the Western Han Dynasty, including the tomb system, the combination of infantry equipment, the organizational equipment of the cavalry, the military system, battle formations, arms, costume craftsmanship, and pottery craftsmanship. Half a century after their excavation, the painted terracotta warriors and horses of the Western Han Dynasty in Yangjiawan, Shaanxi are now displayed at the Xianyang Museum [6] in the city of Xianyang, Shaanxi Province (the location of the painted terracotta warriors and horses of the Western Han Dynasty in Yangjiawan, Shaanxi is shown in Figure 1). 

**Figure 2:**
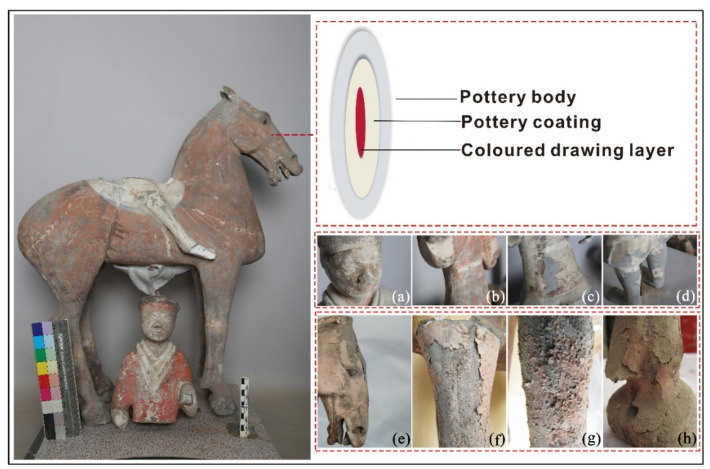
Structure and typical conditions of the painted layer of terracotta warriors and horses from the Western Han Dynasty; (**a**) crisp powder, (**b**) warping, (**c**) cracking, (**d**) shedding, (**e**) warping, (**f**) shedding, (**g**) crisp powder, (**h**) cracking.

The cracks and shedding that occur after painted pottery figurines and pottery horses are unearthed are the most common problems of painted cultural relics (see Figure 2). There are many reasons for their appearance, and some result from a combination of multiple factors [7]. Because these cultural pottery relics were in a highly humid and hypoxic environment for thousands of years before they were unearthed, their environment changed drastically after they were uncovered. When humidity is high, the pigment particles and binder of the painted layer will absorb water and expand; by contrast, when humidity is reduced, the pigment particles and binder of the painted layer will lose water and shrink. Because the shrinkage force of the pigment particles is smaller than that of the binder, the adhesion on the surface of the painted pottery figurines in the pigment layer is reduced, eventually leading to peeling. However, given the material of such pottery, a considerable portion of the cracked and warped painted layer is due to a layer of pottery clothing being made of inorganic materials on the pottery body. Alternatively, because the surface of the pottery body is dense and smooth, the painted layer does not permeate into the pottery body; thus, the adhesion between the painted layer and the pottery clothing or the surface of the smooth and compact pottery body is weakened, resulting in separation. Additionally, due to the aging of the paint layer sizing material, the adhesion of the pigment layer on the pottery clothing surface is reduced, resulting in shedding.

In view of such cracks, the adhesive agent is directly injected into the crack’s inner area, which is meant to improve the adhesion of pigment particles and the pottery matrix. In the past, due to its compatibility with the original cementing material, natural glue (e.g., animal gelatine and peach gum) was used as a reinforcement and binder for the protective treatment of paintings [8]. However, in the second half of the 20th century, research on synthetic polymers opened up new possibilities for restorers due to their decreased sensitivity to relative humidity and visible or ultraviolet light [9]. Currently, polyvinyl alcohol, polyethylene butyrate, ethyl cellulose, polyvinyl acetate, acrylic resins (such as ethyl methacrylate/methyl acrylate copolymer [Paranoid B72]), water-based polyurethane, and fluorocarbon resin are used as synthetic polymers [10,11,12,13,14,15,16,17]. In recent years, FEVE fluoropolymers have become widely used given their excellent weather resistance [18,19].

For the painted terracotta warriors and horses of the Western Han Dynasty, cracks in the painted layer are relatively common. The cracks in the painted terracotta warriors and horses of the Western Han Dynasty can be primarily divided into two types: The first kind is visually identifiable. Generally, a crack bigger than 0.1 mm can be recognized by the human eye. This includes the severity of the painted layer falling off and the exposed pottery body; if the painted layer is cracked, warped, and hollow, it may be in danger of falling off. The second type of crack is at the micron level; it is less than 0.1 mm in size and cannot be seen with the human eye. The gradual development of these micron-level cracks eventually leads to large-scale cracks and the shedding of the painted layer. Herein, we discuss the restoration of such micron-level cracks, about which there have been few reports.

Modern optical inspection technology has developed rapidly, and optical inspection equipment has been widely used in the evaluation, protection, and restoration of artwork [20,21]. An imaging system can be constructed by using imaging principles and a special light source to efficiently assess a piece of art. 

By harnessing ultra-depth-of-field digital microscopy imaging technology [22,23] to build an optical imaging monitoring system operating platform, along with the commercial water-borne FEVE (ZB-F600) as adhesive agent, the micron-level cracks of the terracotta warriors of the Western Han Dynasty were repaired. ZB-F600 water-borne fluoropolymer is a copolymer of trifluoro vinyl chloride, vinyl acetate, allyl alcohol, and fatty acids. It has excellent weather resistance, durability, solubility, transparency, wettability, and adhesion; these characteristics justify the use of ZB-F600 as an adhesive agent in repairing micron-level cracks. To verify this hypothesis, the color difference, adhesion, contact angle, and surface morphology of the painted layer were tested before and after treatment. Meanwhile, in the repair process, the system’s digital microscopic imaging was able to directly obtain information on the microscopic morphology, size, and geometric spaces of the cracks. Thus, a dynamic monitoring and imaging system can be used to assist prosthetists with: real-time visualization of micro-restoration operations, the visual imaging and identification of micron-level cracks, and the video recording of the entire restoration process.

## 2. Status of Preservation

Due to the different environments of the artwork displayed in the museum before being unearthed, the state of preservation of the excavated terracotta warriors and horses is a matter of concern. In addition to weathering, fading, limb breakage, and brokenness, a considerable portion of the painted layer has experienced warping [24,25], shedding [26,27], and cracking, and exhibits a crisp powder. With time, the condition of such artwork has become more serious, and a substantial part of precious information is on the verge of disappearing [28].

## 3. Equipment and Methods

### 3.1. Preparation of Simulated Painted Pottery 

(i) The ceramic pieces were made from the soil collected from Yangjiawan (Xianyang, Shaanxi, China), the excavation area of the painted terracotta warriors and horses of the Western Han Dynasty. A plaster mold was used to shape them into cubes of 12 × 12 × 0.5 cm^3^ by hand and fired at 900 degrees Celsius. The samples were cut into cubes of 3 × 3 × 0.5 cm^3^ using a cutting machine. (ii) To prepare a suitable concentration of pigment paste, a gelatine (TianLli Chemical Reagent Co., Ltd., Hebei, China) solution with a mass concentration of 4% was mixed with mineral pigments, based on a 2:1 mass ratio. The mineral pigments included iron oxide red, cinnabar, malachite green, and ultramarine, made at the JinBiZhai art pigment factory (Beijing, China). Each of the four pigments was applied to the surface of the pottery pieces mentioned above with a brush 0.1–0.2 mm thick, and dried at room temperature. (iii) Six grams of ZB-F600 emulsion (Zhenbang Fluorine PaintCo., Ltd., Dalian, Liaoning, China) were diluted with 100 mL of water, followed by stirring for 20 min. The diluted emulsion was applied to the pottery piece using a brush and dried at room temperature.

### 3.2. Chromatic Aberration Test

VS-450 (X-Rite, Grand Rapids, Michigan, USA) was used to test the chromatic aberration of the simulated painted pottery before and after the reinforcement treatment. The D65 standard light source and the ISO11 467:2000 standard CIE L × a × b × system were used to record the color change in the simulated painted pottery. 

### 3.3. Adhesion Test

**Figure 3:**
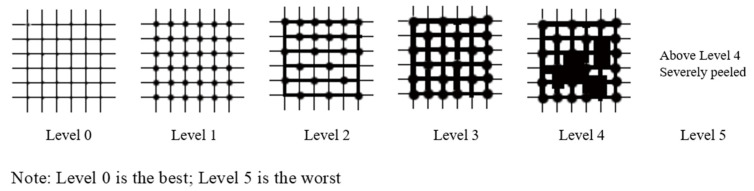
Standard of the adhesion test.

The adhesion of the reinforcing agent was tested via the cross-hatch method. The specific method involves using a blade to make six parallel knife marks on the surface of the painted layer (length: 10–20 mm; the distance between the cut marks is 1 mm), and a cut is made through the entire paint layer. Then, the same six marks are made similar to the former. The cut marks are vertical to form a small square, and a tape is attached to the entire grid and torn at the smallest angle. The test results can be obtained according to the area ratio of the peeled pigment on the painted surface. According to the national standard GB/T1720-1989 for testing, Level 5 is the best, and Level 0 is the worst (see Figure 3).

### 3.4. Contact Angle Test

A contact angle system (OCA 20, Datphysics Instruments GmbH, Filderstadt, Germany) was used under the condition of a fixed water volume of 2 μL. The contact angle of the simulated painted pottery was measured before and after the treatment. The contact angle was the average of three measurements.

### 3.5. Scanning Electron Microscope

A scanning electron microscope (Quant 200, FEI, Hillsboro, OR, USA) was used to observe the surface morphology of the simulated painted pottery before and after treatment. The sample was sputtered with gold, the magnification was 1200, and the acceleration voltage was 20.0 kV.

### 3.6. Thermal Stability Properties 

To evaluate the effect of ZB-F600 on thermal stability, the thermogravimetric analysis (TG) was performed on pure ZB-F600 at a heated rate of 20 °C/min from 25 to 800 °C.

### 3.7. Equipment 

**Figure 4:**
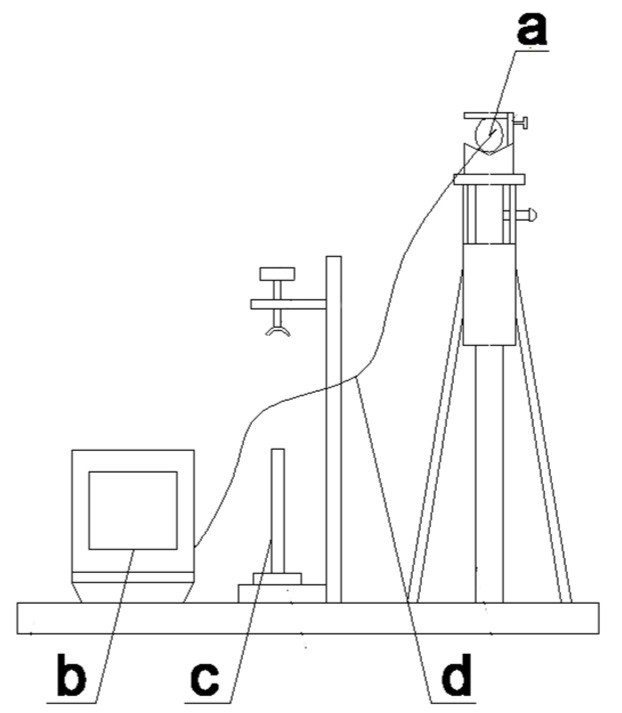
A micron-level crack microscopic dynamic monitoring device; (**a**) camera lens, (**b**) image processor, (**c**) axis of rotation, (**d**) optical fiber.

A microscopic dynamic monitoring device (self-assembly, see Figure 4) [29] for micron-level cracks was used to restore the painted layer of cultural ceramic relics. A super image processor was set on the left side and a tripod was set on the right side of the workbench; furthermore, a lens clamp with the lens was set on the upper end of the tripod. The lens was connected with the super image processor through the optical fiber to form a super depth-of-field microscope. A restoration table was arranged on the workbench between the super image processor and the tripod. The tripod had to be rotated to adjust the lens to make the super image processor clearly display the cracks, to fix the cultural relics, and to close the cracks with the reattachment restoration agent. The utility model device could accurately locate the micron-level cracks in the painted layer, accurately measure the size of the cracks, realize multi-angle and omni-directional observations, and collect images and process videos in real time. The collected data and images were intuitive and precise, and the restored cracks were completely closed and fitted.

The device was equipped with a high-performance ultra-depth-of-field microscope (KEYENCK, VXH600, Shanghai, China) that integrates all functions of condensed observation, recording, and measurement. It can achieve a large depth-of-field observation (20–5000 times), as well as clear and accurate observations of large concave and convex samples that cannot be fully focused on with traditional microscopes. Furthermore, it can locate the position of the micron-level cracks in the painted layer; precisely measure the length, width, and tilt angle of the ceramic cultural relics; and realise the two-dimensional positioning of the target observation. Additionally, the image processor of the ultra-depth-of-field microscope can produce ultra-strong color and has ultra-high fine resolution capabilities. The transmission speed is fast (more than 20 frames per second) and can be divided into screen observations, on-site storage, and recorded images; it can be used for 2D measurements and fast depth synthesis of 2D and 3D displays. Moreover, a simple two-step operation can create fully focused 2D and 3D images and engage in real-time 2D and 3D image splicing.

### 3.8. Methods 

By adjusting the angle of the depth-of-field microscope, cooperating with the movement of the cultural relics installed on the rotating shaft, a multi-angle observation was achieved through the fine adjustment of the adjusting handle of the tripod, eliminating blind spots on the target. The omni-directional observation was achieved by shifting the observation direction. The accurate position, size, and angle of micron-level cracks in the painted layer were seen through monitoring and auxiliary observations of the data, images, and videos.

## 4. Results and Discussion

### 4.1. Chromatic Aberration and Adhesion Changes

The ΔE* values of untreated and treated painted pottery with water-borne fluoropolymer are listed in Table 1. The chromatic aberration of the four treated pieces of painted pottery is less than 1. In general, a ΔE* value of less than 1.5 is undetectable by the human eye [30,31]. Therefore, ZB-F600 water-borne fluoropolymer treatment did not influence the appearance of the painted pottery, which conforms to the principle of ‘maintaining the original appearance of cultural relics’. In addition, the adhesion of the four pigmented layers before and after treatment was tested. The results show that the adhesive force of the painted layer was significantly improved after treatment, indicating that ZB-F600 water-borne fluoropolymer can be used as an adhesive agent in the restoration of painted pottery.

### 4.2. Hydrophobic Performance

**Figure 5:**
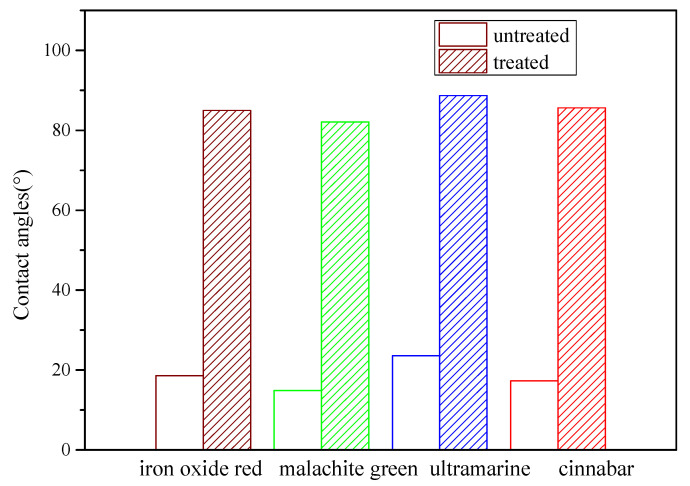
Contact angle of the treated and untreated samples for the four pigmented layers.

Figure 5 shows the contact angle of the treated and untreated samples for the four pigmented layers. The untreated surface was completely hydrophilic. Compared with it, the contact angle of the pieces treated with ZB-F600 water-based fluoride was significantly increased. The hydrophilicity of the samples treated with ZB-F600 water-borne fluoropolymer decreased and had a certain degree of hydrophobicity.

### 4.3. Scanning Electron Microscopy (SEM) Analysis

**Figure 6:**
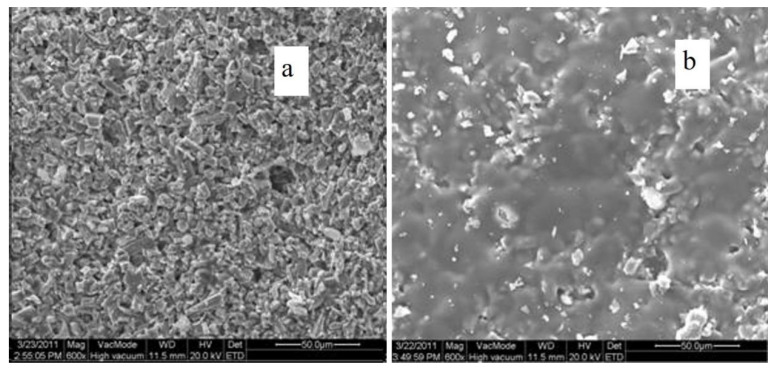
Micro morphology of the untreated (**a**) and treated (**b**) cinnabar samples.

The surface morphologies were analyzed via SEM to further study the reinforcement effects of ZB-F600 water-borne fluoropolymer. The micro morphology of untreated and treated cinnabar samples at a magnification factor of 600 are shown in Figure 6. The results indicate that the untreated cinnabar pigmented layer surface was formed by the different sizes of pigment particles. Meanwhile, there were a lot of pores in the pigmented layer, suggesting poor connection among the pigment particles. Compared with the untreated samples, the pigment particles of the treated samples had a fuzzy edge, which were cemented as a whole because the ZB-F600 water-borne fluoropolymer was cemented and filled some pores. Therefore, ZB-F600 can change the separated pigmented particle microstructure to form a dense film structure and significantly improve the loose nature of the pigmented particles. This dense film structure provided the consolidated pigment layer good water resistance and stability.

### 4.4. Thermal Stability 

**Figure 7:**
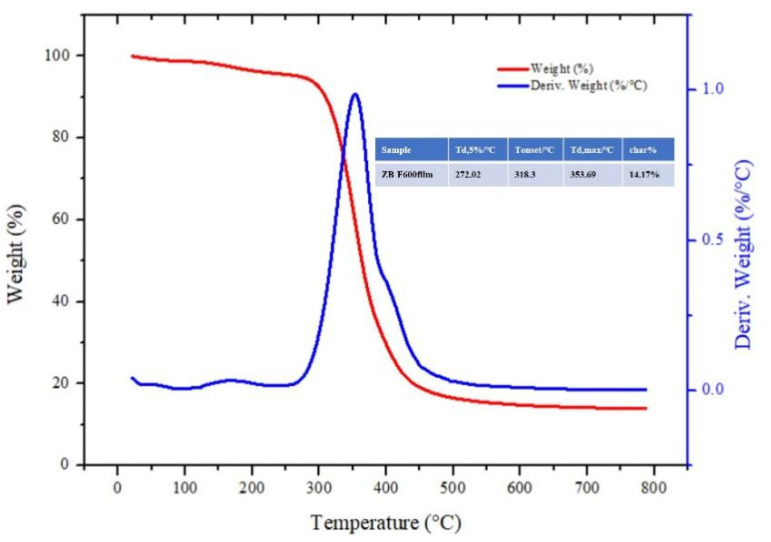
TG and DTG curves of pure ZB-F600 under nitrogen.

The TG and derivative thermogravimetry (DTG) curves of pure ZB-F600 under nitrogen are displayed in Figure 7. There was a 5% loss in temperature (*T*d5%) of the ZB-F600 at 272.0 °C. The onset decomposition temperature, Tonset, was 318.3 °C, and the maximum thermal decomposition temperature, Tmax, was 353.69 °C. These results signal that ZB-F600 has excellent thermal stability.

### 4.5. Monitoring of the Restoration Process: A Case Study of the Painted Terracotta Warriors of the Western Han Dynasty

**Figure 8:**
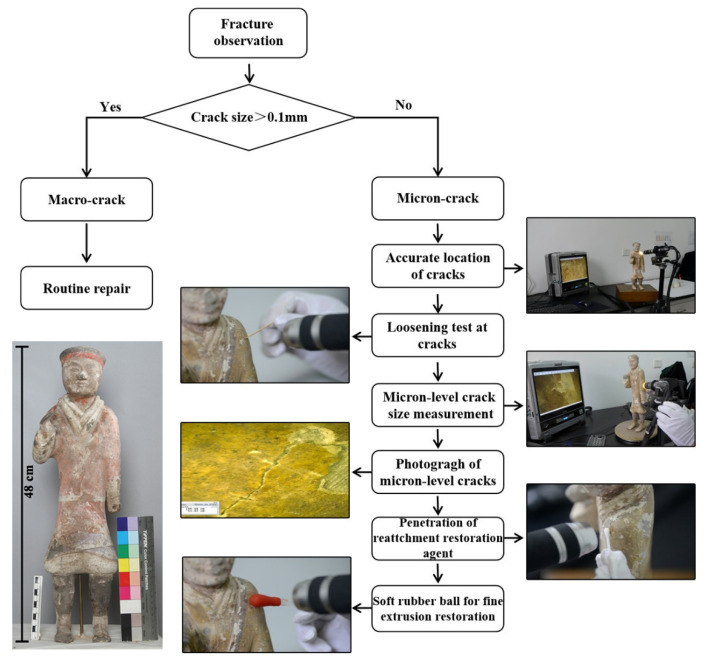
Process of microscopic imaging technology, assisted dynamic monitoring, and restoration.

As presented in Figure 8, for the macroscopic cracks (>0.1 mm), the restoration agent [32] was directly injected. For the micron-level cracks (<0.1 mm), our method involved repair using the ultra-depth-of-field microscope. The dynamic detection system for the micron, meter-level cracks included a repair table, an ultra-deep field optical microscope, and an optical system [33]. The multi-channel image data transmission switching system included a host and terminal. During the restoration process, the cultural relics were fixed on the restoration table. The host connected and controlled the displacement system of the restoration table to adjust its position and angle. The multi-channel image data transmission switching system connected the optical system to the ultra depth-of-field optical microscope through data lines and optical fibers. Then, the multi-channel image data transmission and switching system was connected to the host and the terminal through a data line, and various controls and images of the host were displayed on the terminal. The restoration process was controlled by the host computer to select real-time acquisition of images and to process the video. 

**Figure 9:**
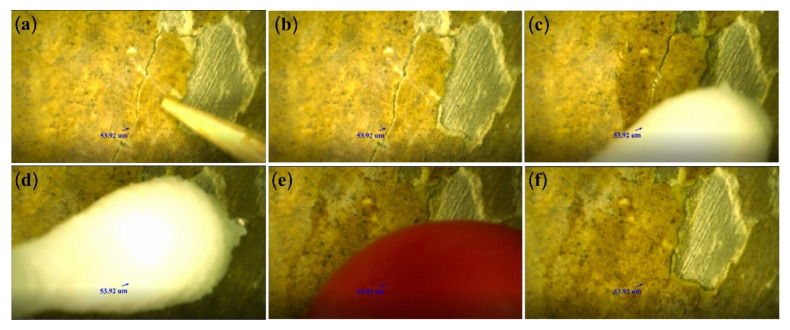
Restoration process of the micron-level cracks. (**a**) Crack location, (**b**) crack measurement, (**c**) penetration of the adhesive agent, (**d**) air is removed from the cracks, (**e**) pressing and restoration, (**f**) after restoration.

The specific repair process is outline below (see Figure 9).

#### 4.5.1. Locating the Cracks

The ceramic relics were installed on the rotating shaft. The lens of the ultra-depth-of-field microscope was adjusted, and the painted layer of the ceramic relics was focused on. The relics were rotated around the axis, and observations were made through the super image processor of the ultra-depth-of-field microscope to locate the micron-level cracks in the painted layer (see Figure 9a). The horizontal or vertical adjusting handle of the tripod was used to rotate the lens of the microscope until the ultra-imaging processor clearly showed the cracks; the adjusting screw was rotated to clamp and fix the ceramic relics with a fixed plate for visual monitoring and auxiliary observation.

#### 4.5.2. Evaluation and Measurement of the Crack Development 

Through the digital microscopic imaging of the system, the information of the microscopic morphology, size, and geometric spaces of the cracks was obtained intuitively, and was used to judge their development in the painted layer to be restored (see Figure 9b) [34,35].

#### 4.5.3. Penetration of the Adhesive Agent

A cotton swab was dipped in the 6% ZB-F600 water-borne fluoropolymer to bond the micro-level cracks under the microscope display screen (see Figure 9c).

#### 4.5.4. Pressing the Cracks in the Painted Layer

**Figure 10:**
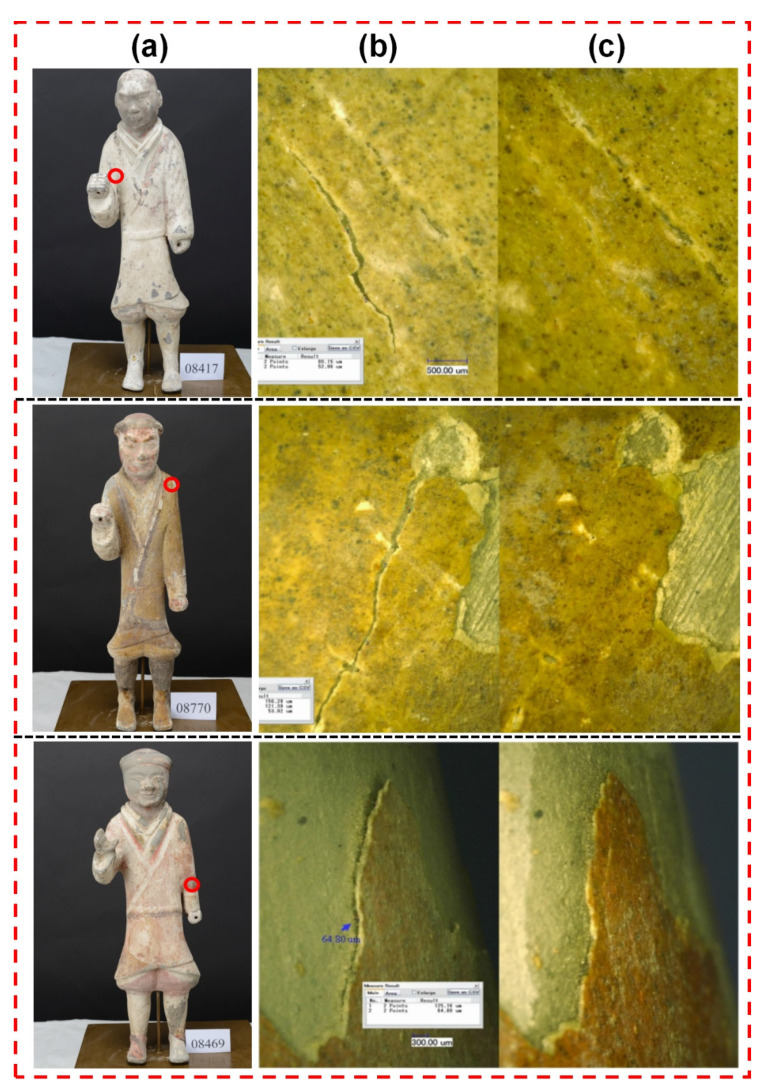
Restoration effect of the painted layer ((**a**) pottery, (**b**) untreated, (**c**) treated).

The painted layer was gently pressed to the cracks with the cotton swab to remove air from inside the cracks (see Figure 9d). Then, the painted layer was squeezed at the cracks with a soft rubber ball; the cracks were fully closed and fitted together (see Figure 9e). The microsystem photography function was used to take pictures of the repaired cracks (see Figure 9f) and the restoration effect is demonstrated in Figure 10.

## 5. Conclusions

This paper examined the painted terracotta warriors and horses from the Western Han Dynasty at the Xianyang Museum, and studied the use of ZB-F600 water-borne fluoropolymer as an adhesive agent to repair micron-level cracks. This was done by employing super depth-of-field digital microscopic imaging technology to build an operating platform for an optical imaging monitoring system. By making simulated painted pottery, the color difference of simulated painted pottery before and after treatment was tested. The ΔE* value of samples after treatment was less than 1, indicating that ZB-F600 water-borne fluoropolymer did not change the original color. The results of the adhesion test show that ZB-F600 water-borne fluoropolymer treatment can improve the adhesion between the painted layer and the pottery matrix. The test results of the contact angle of the untreated and treated painted layer indicate that the contact angle of the treated painted layer significantly improved, which can help preserve the painted cultural relics. The scanning electron microscope results revealed that ZB-F600 water-borne fluoropolymer encased the loose pigment particles and filled in the pores between the particles, forming a dense film on the surface. This suggests that ZB-F600 water-borne fluoropolymer can be used as an adhesive agent for micron-level cracks. The repair process of micron-level cracks was determined using a super depth-of-field, digital microscopic imaging technology. Microscopic imaging can directly obtain the micro-morphology, size, geometry, and spaces of cracks, information that can be used to judge the development of cracks in the coating layer to be repaired. Furthermore, dynamic monitoring and imaging systems can be used to assist prosthetists with visualizing micro-restoration operations in real time, visual fine surgery during restoration, and recording dynamic video of the entire process. The findings imply that the microscopic image-assisted technique has great potential in the dynamic monitoring and restoration of cultural relics; it also improves the visualization method for the repair of micron-level cracks in painted pottery relics.

## Figures and Tables

**Table 1:** Chromatic aberration and adhesion changes of untreated and treated portions of the simulated painted pottery.

Sample	L*	a*	b*	ΔE*	Adhesion
Untreated-iron oxide red	35.56	18.63	16.79		Level 3
Treated-iron oxide red	34.44	18.58	17.01	0.67	Level 1
Untreated-malachite green	71.85	−26.67	10.68		Level 3
Treated-malachite green	73.55	−26.17	10.48	0.71	Level 1
Untreated-ultramarine	35.41	6.99	−42.83		Level 4
Treated-ultramarine	35.54	6.58	−42.01	0.87	Level 1
Untreated-cinnabar	47.95	40.83	22.53		Level 3
Treated-cinnabar	49.06	39.64	21.86	0.76	Level 1

## Data Availability

The datasets used and/or results obtained in the current study are available from the corresponding author upon request.

## Abbreviations

Paranoid B72	ethyl methacrylate/methyl acrylate copolymer
ZB-F600	ZB-F600 water-borne FEVE
2D	two-dimensional
3D	three-dimensional
SEM	scanning electronic microscopy
TG	thermogravimetric analysis
DTG	derivative thermogravimetry
